# A rare case report of gastric cancer complicated by ectopic drainage of the accessory common bile duct into the stomach and corresponding surgical strategies

**DOI:** 10.3389/fsurg.2026.1902083

**Published:** 2026-07-10

**Authors:** Danping Shen, Chen Huang, Chun Zhuang, Chunchao Zhu

**Affiliations:** Renji Hospital, School of Medicine, Shanghai Jiao Tong University, Shanghai, China

**Keywords:** accessory common bile duct, double common bile duct, gastric cancer, radical gastrectomy, Roux-en-Y gastrojejunostomy

## Abstract

**Background:**

Double common bile duct (DCBD) is a rare congenital anomaly, with accessory common bile duct (ACBD) ectopic drainage into the stomach being extremely uncommon and rarely linked to gastric cancer.

**Case report:**

A 48-year-old male with 2-month intermittent retrosternal painand 4-day melena was diagnosed via gastroscopy and pathology. ACBD was intraoperatively misidentified as the accessory left hepatic artery and transected, resulting in bile leakage. He underwent subtotal gastrectomy with Roux-en-Y gastrojejunostomy, D2 lymphadenectomy jejunojejunal end-to-side anastomosis, and choledochojejunostomy, with intraoperative videos, specimen images and pathology reports provided. The reconstructed accessory common bile duct showed unobstructed drainage.

**Conclusion:**

This case highlights the importance of accurate ACBD diagnosis and proper surgical management, and summarizes relevant surgical strategies, offering reference for similar cases.

## 1 Introduction

Double common bile duct (DCBD), a rare congenital anomaly where an accessory common bile duct (ACBD) ectopically drains into the gastrointestinal tract, has long been associated with a spectrum of complications, from biliary lithiasis and cholangitis to more severe conditions like pancreaticobiliary maljunction (PBM) and malignancies (1–5). While the majority of reported DCBD cases involve drainage into the duodenum or pancreatic duct, ectopic drainage into the stomach remains exceedingly uncommon, with only a handful of cases linking this anomaly to gastric cancer. Historical accounts trace the first description of biliary duct duplication to Vesalius in 1543, yet it was not until recent decades that clinical studies, such as Yamashita's review of 47 Japanese cases, highlighted that 25.5% of DCBD patients developed malignancies—specifically noting that gastric cancer frequently arose when the ACBD opened into the stomach (2). Kondo further emphasized this association in 1986, reporting two cases of gastric cancer with ectopic bile duct drainage into the stomach, suggesting a potential carcinogenic role of chronic bile reflux (6). Given the scarcity of such cases and the critical need to understand the relationship between ectopic bile duct drainage and gastric oncogenesis, this report details a unique case of gastric cancer occurring in conjunction with an ACBD draining into the gastric antrum, shedding light on the diagnostic challenges and clinical implications of this rare anomaly.

## 2 Method

Ethics and communication: Informed consent has been obtained. The data collection has been approved by the Ethics Committee of Renji Hospital, Shanghai Jiao Tong University School of Medicine (LY2023-142-B).

## 3 Case report

A 48-year-old male who was admitted to the hospital due to intermittent retrosternal pain accompanied by acid reflux and belching for 2 months, with symptoms worsening for over 20 days and black stools for 4 days. Two months before admission, the patient developed a burning pain behind the sternum without any obvious cause, which was relieved after eating but worsened 3 h after meals. Gastroscopy at an outside hospital suggested low-differentiated adenocarcinoma of the gastric antrum (pathologically confirmed as signet ring cell carcinoma) and chronic non-atrophic gastritis.

The patient has experienced a weight loss of more than 10 kg in the recent month. He has a past medical history of ulcerative colitis for over 10 years, which was treated with traditional Chinese medicine. There is no history of other chronic diseases, surgeries or infectious diseases.

After admission, the patient underwent a comprehensive set of examinations. Chest computed tomography (CT) revealed a calcified lesion in the right lower lung lobe and pleural thickening. Upper abdominal computed tomography angiography (CTA) indicated gastric antral wall thickening (with a range of approximately 5 cm), which was considered suspicious for gastric cancer. Cardiac color Doppler ultrasound showed a decrease in left ventricular diastolic function. MRCP was not performed because it is not part of the routine preoperative workup for gastric cancer resection, which contributed to the failure to identify the double common bile duct (DCBD) preoperatively.

Subsequently, the patient underwent radical gastrectomy, which specifically included subtotal gastrectomy with gastrojejunostomy (Roux-en-Y anastomosis), D2 lymph node dissection, jejunojejunal end-to-side anastomosis, and choledochojejunostomy. Intraoperatively, a tumor was found in the gastric antrum with a diameter of 4 cm, invading the subserosa; perigastric lymphadenopathy was observed, and no distant metastasis was noted.

Postoperative pathology revealed gastric antral signet ring cell carcinoma (measuring 4.3 × 3.2 × 1.1 cm), which invaded the serosal layer. Neural invasion was positive (+), while vascular invasion was negative (-). The surgical margins were negative, and no lymph node metastasis was detected (0/17). Immunohistochemistry results showed: HER2 (0), PD-L1 (CPS=0), Ki67 (30%), and intact expression of mismatch repair proteins (including PMS2 and MLH1). The pathological stage was determined as pT4aN0 (per AJCC 8th edition).

**Figure 1:**
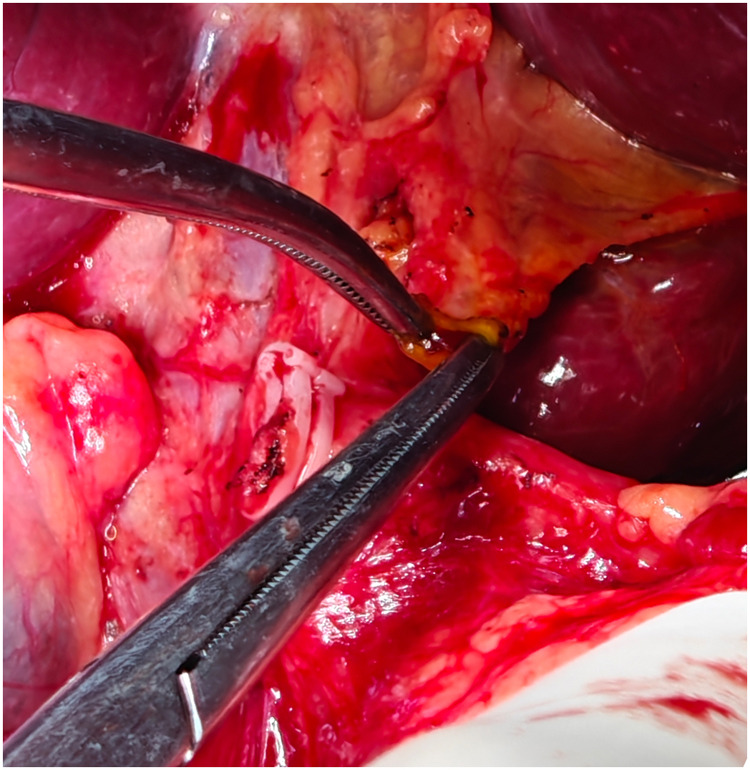
Intraoperative picture.

During the operation, while dissecting the lymph nodes on the lesser curvature of the stomach, a duct was identified. As this duct connected the liver to the stomach, it was initially considered an accessory left hepatic artery and thus transected. After transection, bile leakage was observed from the transected end (Figure 1; Supplementary Video S1).

**Figure 2:**
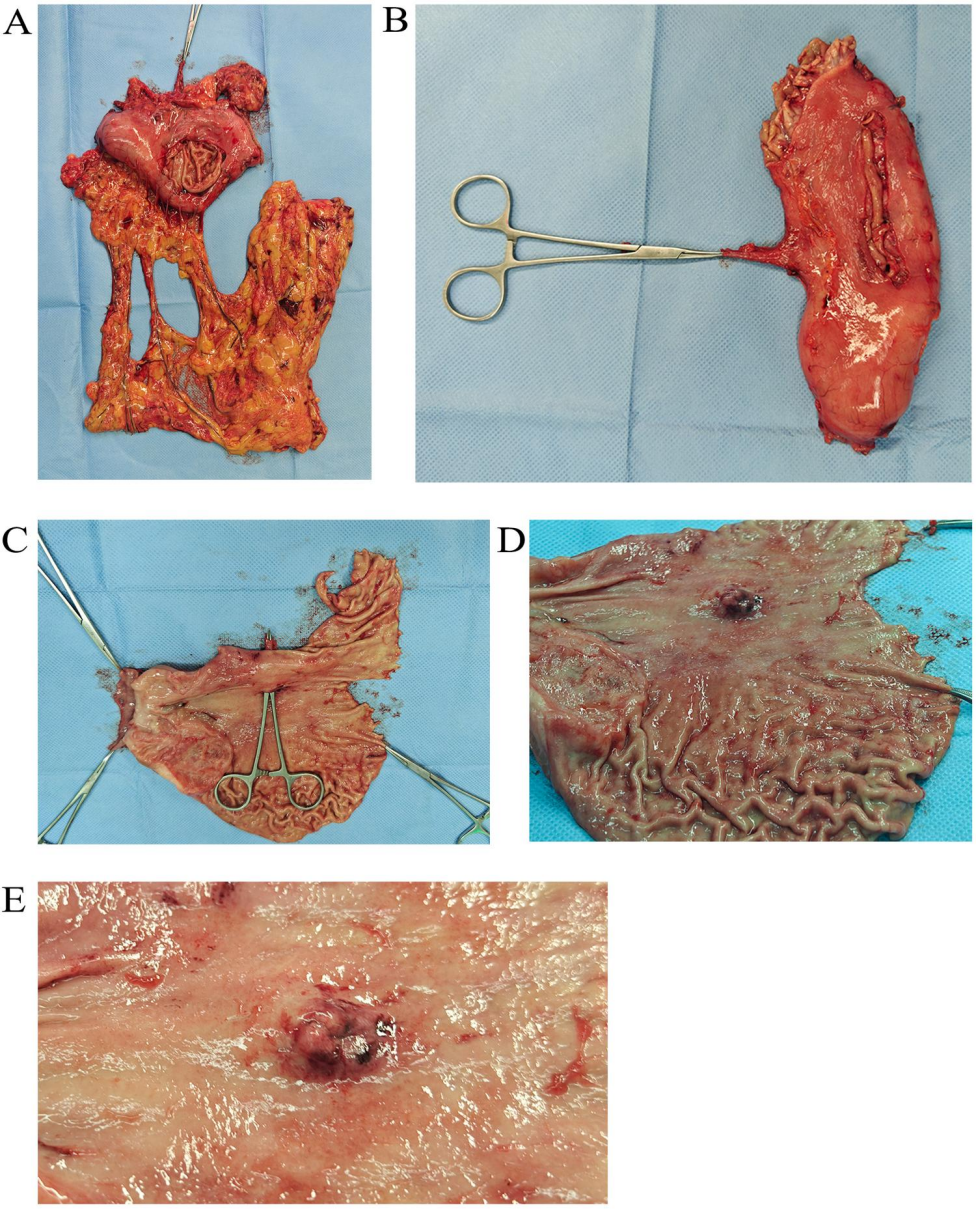
Gross specimen image. **(A)** Resected stomach and lymph nodes from the D2 dissection area; **(B)** the ACBD is located on the lesser curvature of the gastric body; **(C)** the ACBD communicates with the extra-luminal space and opens on the lesser curvature of the gastric body; **(D)** the tumor is located in the gastric antrum, with an approximate distance of 4 cm from the ACBD; **(E)** morphology of the opening of the ACBD.

As shown in the Figure 2, this is the gross specimen of the stomach after standard D2 lymph node dissection, which includes the distal stomach, regional lymph nodes, and the ACBD. After we trimmed the lymph nodes to clearly expose the location of the ACBD, a hemostat was used to examine the patency of the ACBD. After opening the stomach, the distance between the ACBD and the tumor was approximately 4 cm.

**Figure 3:**
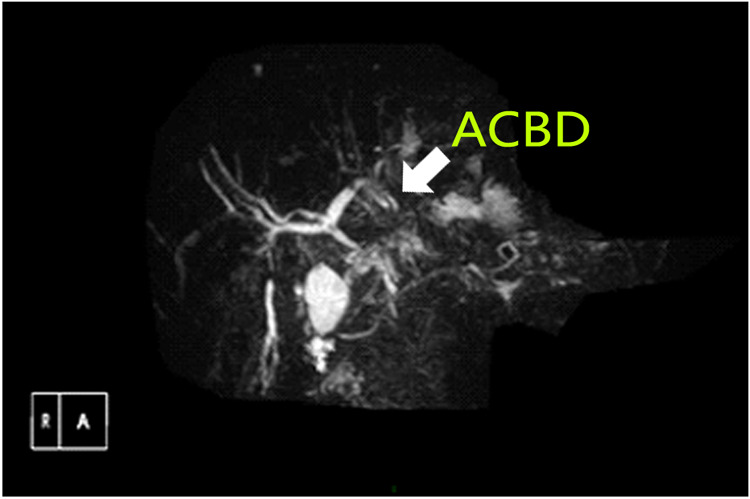
MRCP image after digestive tract reconstruction (3 months postoperatively).

The patient remained nil per os for 5 days postoperatively, started oral water on postoperative day 6, advanced to liquid diet on postoperative day 7, and was discharged on postoperative day 10. No complications such as fever, infection, anastomotic fistula or bile leakage were noted during hospitalization. The reconstructed accessory common bile duct drains into the small intestine, with unobstructed drainage. MRCP performed 3 months after surgery in this case showed “unobstructed drainage of the reconstructed ACBD” without anastomotic obstruction or cholestasis (Figure 3).

## 4 Discussion

### 4.1 Differentiation of the ACBD draining into the stomach from the accessory left hepatic artery

In the surgical procedure for gastric cancer complicated with ectopic drainage of the ACBD, the intraoperative differentiation between ACBD and the accessory left hepatic artery is a core step to avoid iatrogenic injury. Due to their highly overlapping anatomical locations (both are commonly found on the lesser curvature of the stomach and in the hepatogastric ligament area), clinical misjudgment is likely to occur. The intraoperative experience of this case—initially, ACBD was misidentified as the accessory left hepatic artery and transected, and the judgment was corrected only after bile exuded from the transection end—provides direct practical reference for clinical differentiation. The key points for differentiation can be elaborated from two aspects: preoperative imaging evaluation and intraoperative real-time judgment.

From the perspective of preoperative evaluation, the targeted characteristics of different imaging examinations can assist in the initial differentiation of their properties: Magnetic Resonance Cholangiopancreatography(MRCP) can clearly display the biliary duct contour, origin (mostly communicating with the intrahepatic biliary system), and drainage endpoint of ACBD (in this case, it was the lesser curvature of the gastric body); in contrast, contrast-enhanced abdominal CT or CT angiography (CTA) can clearly identify the vascular course of the accessory left hepatic artery (mostly originating from the celiac trunk, left gastric artery, or splenic artery and supplying blood to the hepatic parenchyma) as well as its blood flow signals (4, 7). In this case, only chest CT, upper abdominal CTA, and gastroscopy were performed preoperatively, while MRCP was not conducted. This resulted in the failure to detect the anatomical abnormality of ACBD before surgery, which indirectly increased the risk of intraoperative misjudgment. However, MRCP is not a routine examination before gastric cancer surgery, so its significance for differentiation is limited.

Intraoperative immediate differentiation should focus on the anatomical and functional differences between the two, and the judgment can be achieved through three steps.

The first step is palpation and blood flow detection. As an arterial vessel, the accessory left hepatic artery can be felt with a distinct pulsation during palpation, and a continuous blood flow signal can be detected using an ultrasonic Doppler flowmeter. In contrast, the ACBD is a biliary structure with no pulsation, and no blood flow signal is detected by Doppler. In this case, blood flow detection was not performed first during the operation, and the duct was mistakenly identified as an artery directly based on its anatomical location of “connecting the liver to the stomach”, which indicates the necessity of this step.

The second step is differentiation by puncture and fluid aspiration. A fine needle is used to puncture the suspected duct: if pale yellow clear fluid (bile) is aspirated, it is an ACBD; if bright red blood is aspirated, it is the accessory left hepatic artery. Although preoperative puncture was not conducted in this case, the manifestation of bile exuding from the cross-section after transection is essentially consistent with the differentiation logic of puncture and fluid aspiration, and can be used as a remedial differentiation method.

The third step is tracking the course and terminal. The terminal course of the accessory left hepatic artery is the liver parenchyma (with a clear blood supply target), while the terminal course of the ACBD is the gastrointestinal mucosa (in this case, it opens on the lesser curvature of the gastric body). During the operation, by patiently tracking the direction of the duct's course, the two can be distinguished from the differences in drainage/blood supply targets (4). In this case, the course feature of the duct “connecting the liver to the stomach” actually conforms to the anatomical characteristics of ACBD, i.e., “intrahepatic origin - gastrointestinal drainage”. It is a lesson to be learned that this detail was overlooked due to empirical judgment.

### 4.2 Decision-Making on transection versus preservation of the anomalous communicating bile duct (ACBD)

The decision of transection or preservation of the ACBD (Anomalous Communicating Bile Duct, note: if “ACBD” refers to another anatomical structure, please adjust the full name according to the actual medical context) shall take “ensuring radical tumor resection and avoiding postoperative complications” as the core principle, and be comprehensively determined by integrating the extent of tumor invasion, the anatomical location and functional properties of the ACBD. The decision-making process of this case (eventually choosing to transect the ACBD) can serve as a typical reference, with the specific decision-making logic as follows.

#### 4.2.1 Applicable scenarios for preserving the ACBD

The preservation of the ACBD may be considered only when it meets the three major criteria of “no lesions, no impact on radical tumor resection, and controllable long-term risks”: First, the ACBD itself has no organic lesions (such as no bile duct stricture, calculus, or inflammation), the lumen is confirmed unobstructed during intraoperative exploration, its communication with the main common bile duct is normal, and there is no dependence on its independent drainage function (8); Second, the ACBD is at a relatively long distance from the tumor (a distance of > 5 cm is generally recommended) and is not located in the lymph node dissection area (e.g., far from the dissection range of the lesser curvature and greater curvature of the stomach); its preservation should not affect the tumor resection margin (which must meet the R0 resection standard) or the thoroughness of lymph node dissection (9); Third, there is no risk of carcinogenesis at the drainage endpoint of the ACBD. For example, if the ACBD drains into the duodenum (rather than the stomach), it can avoid the long-term irritation of gastric mucosa caused by bile reflux. In this case, the ACBD drains into the stomach; literature suggests that this drainage pattern is associated with carcinogenesis induced by chronic bile reflux (10), and even if preserved, there is a risk of long-term carcinogenesis, so such situations need to be excluded.

#### 4.2.2 The necessity of ACBD transection

The decision to transect the ACBD in this case was primarily based on the following three core considerations: First, it would interfere with radical tumor treatment procedures. The ACBD was located on the lesser curvature of the stomach, a critical region for D2 lymph node dissection in gastric cancer (requiring dissection of lymph node stations 1 and 3 on the lesser curvature of the stomach). Preserving the ACBD would potentially hinder the manipulation of dissection instruments, leading to incomplete lymph node dissection, which was inconsistent with the surgical goal of “radical gastrectomy with D2 lymphadenectomy” in this case. Second, the long-term risk of carcinogenesis was uncontrollable. The ACBD had ectopic drainage into the stomach, and long-term bile reflux has been confirmed in the literature (e.g., as reported by Kondo in 1986, mentioned in the text) to be associated with the development of gastric cancer (1). Since the patient had been diagnosed with signet ring cell carcinoma of the gastric antrum, preserving the ACBD might expose the residual stomach to persistent bile irritation, increasing the risk of metachronous gastric cancer in the residual stomach. Third, there was a need for remediation after an intraoperative misjudgment. In the initial stage of the operation, the ACBD was mistakenly identified as the accessory left hepatic artery and transected; although this was an operational error, bile leakage was observed from the transected section after the procedure. If forced repair and preservation were attempted at this point, it would require additional surgical time and might increase the risk of postoperative bile leakage due to ductal injury (11). Considering the requirement for radical tumor treatment, the decision was ultimately made to maintain the transection plan, with subsequent reconstruction to address the drainage issue.

It is particularly emphasized that intraoperative decisions require dynamic adjustment: if the anatomy of the ACBD has been clearly identified via MRCP preoperatively, a “preservation/transection” plan should be formulated in advance; in cases of unexpected intraoperative misjudgment (such as the one in this case), blind continuation of the operation is not advisable. Instead, it is necessary to first clarify the nature of the duct through methods such as blood flow detection and puncture aspiration, and then determine whether to perform transection based on the requirement for radical tumor treatment. This avoids sacrificing the efficacy of tumor treatment for the sake of “preservation for preservation's sake” or causing unnecessary damage due to “blind transection”.

### 4.3 Whether to perform reconstruction after transection

Whether to perform reconstruction after transection of the ACBD depends primarily on the “integrity of biliary drainage”—specifically, whether there is an obstacle to bile excretion or a risk of peritoneal infection after transection. In this case, bile oozed from the transected end of the ACBD after transection, and reconstruction was ultimately chosen. The decision-making logic for this can be divided into two scenarios: “reconstruction is mandatory” and “reconstruction may be temporarily unnecessary,” and a comprehensive judgment must be made based on the intraoperative findings and postoperative monitoring.

#### 4.3.1 Clinical indications for mandatory reconstruction

Reconstruction must be performed after transection of the ACBD in the following two scenarios; otherwise, severe postoperative complications will occur. First, when bile leakage occurs after transection. For instance, in this case, continuous bile oozing from the transected end of the ACBD after transection indicated continuous bile flow within the duct (as the ACBD is an important drainage branch of the intrahepatic bile ducts). Without reconstruction, bile would accumulate in the abdominal cavity, causing peritoneal infection, peritonitis, or even sepsis (11). In this case, the reconstruction plan was initiated immediately upon detection of bile leakage during the operation, laying the foundation for postoperative recovery. Second, when the ACBD assumes a key drainage function. If intraoperative ultrasound after transection or postoperative imaging (such as MRCP) indicates intrahepatic bile duct dilation, it suggests that the ACBD is the main channel for intrahepatic bile excretion (with possible stenosis or insufficient drainage of the main common bile duct). Failure to perform reconstruction will lead to complications such as cholestatic hepatitis and jaundice. In this case, MRCP at 3 months postoperatively showed “unobstructed drainage after ACBD reconstruction,” which indirectly confirmed that the ACBD had undertaken certain drainage functions preoperatively and further supported the necessity of reconstruction.

#### 4.3.2 Strict conditions for temporary Non-reconstruction

Temporary non-reconstruction may be considered only when the ACBD meets the criteria of “being a small branch, having weak drainage function, and with unobstructed main biliary tract”, but enhanced postoperative monitoring is required. First, when the diameter of the ACBD is less than 3 mm (12, 13), intraoperative exploration shows that the main common bile duct is unobstructed (without stenosis or stones), there is no dilation of intrahepatic bile ducts, and no bile oozing from the transected end after transection, which indicates that its contribution to biliary drainage is minimal. For example, some small accessory bile duct branches, when transected, do not affect the overall bile excretion. Second, when the patient's general condition is extremely poor and cannot tolerate prolonged reconstruction surgery (such as complicated with severe cardiopulmonary failure or septic shock), temporary non-reconstruction can be performed on the premise of close monitoring of postoperative liver function (bilirubin, alkaline phosphatase) and abdominal ultrasound. If signs of cholestasis appear (such as elevated bilirubin or bile duct dilation), secondary reconstruction should be performed.

In this case, after transection of the ACBD, there was both bile leakage and the ACBD undertook certain drainage functions. Additionally, the patient's general condition was acceptable (only with left ventricular diastolic dysfunction and no severe underlying diseases), meeting the dual indications for “mandatory reconstruction”. Therefore, simultaneous reconstruction was ultimately performed, and no complications such as cholestasis or peritoneal infection occurred postoperatively, which verified the rationality of this decision.

### 4.4 The method for reconstructing

The method for reconstructing the ACBD after transection should be selected based on the surgical scenario (whether concurrent radical gastrectomy is performed), the diameter of the ACBD, and its anatomical location. The core goal is to “achieve unobstructed bile drainage and prevent bile reflux into the stomach”. In this case, concurrent radical gastrectomy (including subtotal gastrectomy plus Roux-en-Y gastrojejunostomy) was performed, and choledochojejunostomy was ultimately chosen. This method is highly compatible with the surgical scenario; its selection basis and advantages can be analyzed in detail in the context of this case, and the applicable boundaries of other reconstruction methods also need to be clearly defined.

This method is applicable to patients undergoing concurrent radical gastrectomy (especially those requiring Roux-en-Y digestive tract reconstruction) (14). The key operational point is to perform an end-to-side anastomosis between the transected end of the ACBD and the jejunal loop (in this case, combined with the jejunal loop used for Roux-en-Y anastomosis). Its advantages are reflected in three aspects: first, it is compatible with the concurrent surgical process. The Roux-en-Y gastrojejunostomy in radical gastrectomy has already mobilized the jejunal loop, eliminating the need for additional mobilization of digestive tract organs. This jejunal loop can be directly used for biliary anastomosis, which reduces the surgical time and trauma. The surgical plan in this case (subtotal gastrectomy + Roux-en-Y anastomosis + choledochojejunostomy) achieved the integrated operation of “tumor radical resection + biliary reconstruction” (14); second, it avoids the risk of bile reflux. The ACBD originally drained into the stomach (as in this case), and long-term bile reflux is one of the carcinogenic factors. Choledochojejunostomy drains bile into the jejunum, which conforms to the physiological drainage pathway of “bile-intestine” and fundamentally interrupts the pathological cycle of “bile reflux - gastric mucosal injury”, highly aligning with the patient's need for preventing residual gastritis and residual gastric cancer after surgery; third, it has high drainage stability. The peristaltic function of the jejunal loop can promote bile emptying, and the anastomotic tension is low (the jejunum has high mobility, allowing adjustment of the anastomotic angle according to the location of the ACBD), resulting in a low incidence of postoperative anastomotic stenosis and bile leakage. MRCP performed 3 months after surgery in this case showed “unobstructed drainage of the reconstructed ACBD” without anastomotic obstruction or cholestasis, verifying the effectiveness of this method.

In clinical practice, other reconstruction methods should be selected based on the patient's specific conditions, but their scope of application must be clearly defined. First, choledochoduodenostomy is only applicable to patients where the transected end of the ACBD is close to the duodenum and no subtotal gastrectomy has been performed (e.g., patients with early gastric cancer undergoing endoscopic resection with concurrent management of the ACBD). This method is simple to operate but carries the risk of bile reflux into the stomach (especially in patients with a preserved whole stomach). Moreover, it is not suitable for scenarios like the one in this case, where “the position of the duodenum changes after subtotal gastrectomy,” so it was not adopted. Second, choledocho-remnant gastric anastomosis is only used temporarily in extreme emergency situations (e.g., unstable vital signs in the patient requiring rapid completion of reconstruction). Although this method involves a short operative distance, bile flows directly into the remnant stomach, which exacerbates mucosal injury of the remnant stomach. This contradicts the goal of “avoiding bile reflux” in this case, and it may increase the risk of remnant gastric cancer in the long term, so it is rarely used as a routine option in clinical practice.

In conclusion, choledochojejunostomy is the optimal choice for ACBD reconstruction in this case due to its compatibility with the concurrent radical gastrectomy process, ability to avoid the risk of bile reflux, and stable drainage. It also provides a reference paradigm for the reconstruction method in similar cases of “gastric cancer combined with ectopic drainage of ACBD into the stomach”.

## Data Availability

The original contributions presented in the study are included in the article/Supplementary Material, further inquiries can be directed to the corresponding author.

## Supplementary material

Supplementary Video S1 Intraoperative video.

## References

[1] Doan TT Pham DT Van Nguyen C Tran TT Van Nguyen H. Double common bile duct - a rare case report and review of literature. Int J Surg Case Rep. (2025) 133:111519. 10.1016/j.ijscr.2025.111519 40527006 PMC12213094

[2] Yamashita K Oka Y Urakami A Iwamoto S Tsunoda T Eto T. Double common bile duct: a case report and a review of the Japanese literature. Surgery. (2002) 131(6):676–81. 10.1067/msy.2002.124025 12075184

[3] Ma XS Feng MF Ke S Yang L. Duplicate gallbladders misdiagnosed as residual cholecystitis: a case report and review of the literature. Medicine (Baltimore). (2024) 103(51):e40367. 10.1097/MD.0000000000040367 39705461 PMC11666152

[4] Hashimoto K Ihara T Maruyama Y Tomisaki S Harada H. An operative case of gastric cancer with ectopic bile duct drainage in the lesser curvature of the stomach. Surg Case Rep. (2024) 10(1):67. 10.1186/s40792-024-01862-5 38509272 PMC10954595

[5] Lee JH Yu JS Park MS Yoon DS Yang SW. Mr cholangiography of accessory bile duct connected to the stomach. AJR Am J Roentgenol. (2007) 189(6):W344–7. 10.2214/AJR.05.1570 18029847

[6] Kondo K Yokoyama I Yokoyama Y Harada A Nagayo T. Two cases of gastric cancer-bearing double choledochus with ectopic drainage into the stomach. Cancer. (1986) 57(1):138–41. 10.1002/1097-0142(19860101)57:1<138::aid-cncr2820570128>3.0.co;2-m 3940613 10.1002/1097-0142(19860101)57:1<138::aid-cncr2820570128>3.0.co;2-m

[7] Welle CL Miller FH Yeh BM. Advances in mr imaging of the biliary tract. Magn Reson Imaging Clin N Am. (2020) 28(3):341–52. 10.1016/j.mric.2020.03.002 32624153

[8] Frey S De Mathelin P Bachellier P Addeo P. Aberrant left gastric vein: what should surgeons know? Surg Radiol Anat. (2022) 44(9):1247–50. 10.1007/s00276-022-03009-3 36068438

[9] Kuroda K Miki Y Kasashima H Yoshii M Fukuoka T Tamura T. Optimal extent of lymph node dissection for high-risk gastric cancer stratified by a national clinical database risk calculator. World J Surg. (2024) 48(5):1198–208. 10.1002/wjs.12117 38391091

[10] Shi X Chen Z Yang Y Yan S. Bile reflux gastritis: insights into pathogenesis, relevant factors, carcinomatous risk, diagnosis, and management. Gastroenterol Res Pract. (2022) 2022:2642551. 10.1155/2022/2642551 36134174 PMC9484982

[11] Feng F Cao X Liu X Qin J Xing Z Duan J. Two forms of one complication: late erosive and nonerosive postpancreatectomy hemorrhage following laparoscopic pancreaticoduodenectomy. Medicine (Baltimore). (2019) 98(30):e16394. 10.1097/MD.0000000000016394 31348239 PMC6709069

[12] Wong DK. The accessory bile duct of luschka and bile leakage in laparoscopic cholecystectomy. Hawaii Med J. (1994) 53(6):164–5. 8077109

[13] Justaniah A Abughararah MZ Ahmad N Ashour M Alqarni H. Percutaneous management of hepatic duct injury using extra-anatomic biliary catheters. Cureus. (2023) 15(2):e35012. 10.7759/cureus.35012 36938281 PMC10021372

[14] Androulakis J Colborn GL Skandalakis PN Skandalakis LJ Skandalakis JE. Embryologic and anatomic basis of duodenal surgery. Surg Clin North Am. (2000) 80(1):171–99. 10.1016/s0039-6109(05)70401-1 10685148

